# Substance Use Among Female Sex Workers in Low- and Middle-Income Countries: A Systematic Review and Meta-Analysis

**DOI:** 10.1192/bjo.2024.116

**Published:** 2024-08-01

**Authors:** Tara Beattie, Alicja Beksinska, Molly Fitzgerald, Oda Karlsen, Tanya Abramsky

**Affiliations:** 1 Department of Global Health and Development, London School of Hygiene and Tropical Medicine, London, United Kingdom; 2 Health Equity and Innovation, Juxta Health LLC, Washington, USA

## Abstract

**Aims:**

This systematic review aimed to quantify the prevalence of substance use among female sex workers (FSWs) in low- and middle-income countries (LMICs).

**Methods:**

Design: The review protocol was registered with PROSPERO (CRD42021242048). We searched Ovid, PubMed and Web of Science databases for peer-reviewed, quantitative studies from inception to 6th March 2023. Study designs included: cross-sectional, case–control, cohort study, case series analysis, or experimental studies. Study quality was assessed using the Centre for Evidence-Based Management (CEBM) Critical Appraisal Tool.

Setting: FSWs in LMICs.

Participants/Inclusion criteria: any measure of prevalence or incidence of substance use (not alcohol or tobacco) among FSWs aged 18+ years.

Measurements: A narrative synthesis was conducted across all studies meeting the inclusion criteria. Pooled prevalence estimates for ‘ever’ and ‘recent’ drug use were calculated using a random effects model.

**Results:**

3135 papers were identified; 161 papers reporting on 102 studies with 167,333 FSWs from 39 LMICs met the inclusion criteria. 26 studies scored high, 61 scored moderate, and 15 scored in the lower quality range. Only 4/102 studies used a validated measurement tool to assess levels of substance use dependence. The mean age of study participants was 28.9 years (SD 7.7). The pooled prevalence for recent (past month to past year) substance use among FSWs in LMICs is: illicit drug use 29% (95% CI: 14–34%), cannabis 20% (95% CI: 8–30%), cocaine 21% (95% CI: 9–32%), amphetamine type stimulants 19% (95% CI: 12–26%), opioids 8% (95% CI: 4–12%), sedatives and sleeping pills 6% (95% CI: 0–12%), inhalants 4% (95% CI: –4–12%), hallucinogens 0% (95% CI: 0–0%), and recent drug use during sex work 42% (95% CI: 15%–68%). Only 5/102 studies reported a substance use intervention. Key study limitations include the lack of a validated measurement tool by most studies (96%) meaning it was not possible to distinguish between any drug use vs. harmful drug use. The criminalisation of drug use may have led to under-reporting and an underestimate of true substance use prevalences.

**Conclusion:**

FSWs in LMICs report a high prevalence of recent drug use – including during sex work – with cannabis, cocaine and amphetamine type stimulants the most commonly used. There is an urgent need for effective low-cost substance use interventions. Future studies should use validated substance-use measurement tools to assess the burden of substance use disorders.

